# Analysis of Soret and Dufour effects on radiative heat transfer in hybrid bioconvective flow of carbon nanotubes

**DOI:** 10.1038/s41598-024-62647-2

**Published:** 2024-05-25

**Authors:** Azad Hussain, Saira Raiz, Ali Hassan, Ahmed M. Hassan, Hanen Karamti, Gabriella Bognár

**Affiliations:** 1 Department of Mathematics, University of Gujrat, Gujrat, 50700 Pakistan; 2 Department of Mechanics and Aerospace Engineering, Southern University of Science and Technology, Shenzhen, Guangdong China; 3 Faculty of Engineering, Future University in Egypt, Cairo, Egypt; 4 Department of Computer Sciences, College of Computer and Information Sciences, Princess Nourah bint Abdulrahman University, P.O. Box 84428, 11671 Riyadh, Saudi Arabia; 5 Institute of Machine and Product Design, University of Miskolc, Miskolc-Egyetemvaros, 3515 Hungary

**Keywords:** Prandtl hybrid nanofluid, Soret and Dufour, Bioconvection, Motile microorganisms, magneto-hydrodynamic, Thermal radiation, SWCNT, MWCNT, Mechanical engineering, Applied mathematics

## Abstract

Numerous heat transfer applications, such as heat exchangers, solar trough collectors, and fields including food processing, material research, and aerospace engineering, utilize hybrid nanofluids. Compared to conventional fluids, hybrid nanofluids exhibit significantly enhanced thermal conductivity. The aim of this work is to explore flow and heat transmission features under of magneto-hydrodynamic bioconvective flow of carbon nanotubes over the stretched surface with Dufour and Soret effects. Additionally, comparative dynamics of the carbon nanotubes (SWCMT − MWCNT/C_2_H_6_O_2_ with SWCMT − MWCNT/C_2_H_6_O_2_ − H_2_O) flow using the Prandtl fluid model in the presence of thermal radiation and motile microorganisms has been investigated. Novel feature Additionally, the focus is also to examine the presence of microorganisms in mixture base hybrid nanofluid. To examine heat transfer features of Prandtl hybrid nanofluid over the stretched surface convective heating is taken into consideration while modeling the boundary conditions. Suitable similarity transform has been employed to convert dimensional flow governing equations into dimensionless equations and solution of the problem has been obtained using effective, accurate and time saving bvp-4c technique in MATLAB. Velocity, temperature, concentration and microorganisms profiles have been demonstrated graphically under varying impact of various dimensionless parameters such as inclined magnetization, mixed convection, Dufour effect, Soret effect, thermal radiation effect, and bioconvection lewis number. It has been observed that raising values of magnetization (0.5 ≤ M ≤ 4), mixed convection (0.01 ≤ λ ≤ 0.05) and inclination angle (0° ≤ α ≤ 180°) enhance fluid motion rapidly in Ethylene glycol based Prandtl hybrid nanofluid (SWCMT − MWCNT/C_2_H_6_O_2_) when compared with mixture base working fluid of carbon nanotubes SWCMT − MWCNT/C_2_H_6_O_2_ − H_2_O). Raising thermal radiation (0.1 ≤ Rd ≤ 1.7) and Dufour number (0.1 ≤ Du ≤ 0.19) values improves temperature profile. Moreover, a good agreement has been found between the current outcome and existing literature for skin friction outcomes.

## Introduction

Hybrid nanofluids means presence of two nano-meter sized nanoparticles in the working fluid. This idea is typically used to boost thermal conductivity of the formed fluid, it has been observed that hybrid nanofluids posses far greater thermal conductivity and ability to transfer heat in numerous application such as food processing, cooling process and heat exchangers effectively when compared with nanofluids^1–5^. Carbon nanotubes are the nanometre sized single layer and multi-layer structures^6–10^ which play in applications namely; solar trough collectors^11,12^, solar energy^13,14^ and heat transfer in rotating flows^15,16^. In the recent years, the advancement in the concept of fluids has pushed the researchers to further innovate the existing concept on the nano and hybrid nanofluids. Scientist around the world are now under taking the suspension of three nanoparticles instead of two to further enhance thermal conductivity of formed liquids and address the concerning issue of efficient heat transfer and enhancement, this generally new kind of fluids are termed as ternary hybrid nanofluids^17^.

Gul et al.^18^ discussed hybrid nanofluid for heat transfer applications in a porous cavity. Nasir et al.^19^ investigated the hybrid nanofluids with nonlinear chemical reaction for the flow and heat transfer attributes. Nasir^20^ examined water and ethylene glycol flow for entropy generation using Darcy-forchheimer flow. Dawar et al.^21^ studied the Non-Newtonian fluid with hybrid nanofluids using the alumina and copper nanoparticles. Mishra^22^ used bio-active mixers and chemical reaction to investigate the hybrid nanofluids for chemically reacting jet flow. Raizah et al.^23^ explored Hall current and heat source effects on hybrid nanofluids. Alrabaiah et al.^24^ studied Darcy-Forchheimer flow using radiative hybrid nanofluids over slandering sheet. Bilal^25^ explored the hybrid nanofluids numerically using the second order chemical reaction over slandering surface. Algehyne et al.^26^ explored ternary hybrid nanofluids using the variable diffusion and non-Fourier law. Nasir^27^ examined ternary hybrid nanofluids using the magnetic dipole effect and thermal radiation for the heat transfer feature. Alnahdi^28^ studied ternary hybrid nanofluids using the Casson fluid for medication application in convergent/divergent channel.

The effects described by Soret and Dufour perform a crucial role in fluid mechanics, especially essential for fluids with higher temperature and concentration variations. In a starting point homogeneous mixture exposed to a temperature gradient, the thermo-diffusion (Soret) influence correlates with species differentiation, the diffusion-thermo (Dufour) affects the heat flow created by a concentration gradient. As the result, concentration and temperature will affect the species’s ability to diffuse and expend energy. Pal and Mondal^29^ analyzed viscous non-Darcian’s MHD mixed convection mass and heat transport with Soret and Dufour impacts. Makinde^30^ explored the MHD convection with Soret and Dufour effect over porous vertical plate. Reddy and Rao^31^ investigated mass and heat flow across cylindrical annulus with quadratically varying temperature using FEM under Soret and Dufour effects. Chamkha and Rashad^32^ discussed unsteady mass and heat transmission flow under the influence of Soret and Dufour impacts. Researcher have examined Soret and Dufour effects over different geometries with distinct assumptions and in miscellaneous flow regimes^33,34^.

When bacteria travel unpredictably like a single cell structures, a phenomenon known as bioconvection occurs. The severity of atypical layering brought on by microorganisms creeping upward is what causes convection. Algehyne^35^ studied actication energy and heat source effect with gyrotactic microorganism in hybrid nanofluid over a Riga plate. Raizah^36^ investigated motile microorganism hybrid nanofluid across a circular cylinder with sinusoidal radius. Algehyne^39^ examined nanofluid flow in the presence of motile microorganism over vertical permeable surface. Hussain^40^ explored bioconvective flow in the presence of microorganism. Alharbi^41^ described bioconvection due to gyrotactic microorganisms in hybrid nanofluid with magnetic nanoparticles and with chemical reaction through porous stretching sheet. Mahdy et al.^42^ studied Non-Newtonian bioconvection flow with microorganisms over stretching surface. Chandra and Das^43^ explored vertically inclined surface in presence gyrotactic microorganisms employing machine learning. Gyrotactic microorganisms have been examined extensively in the presence of different body force effects and under distinct flow regimes^44–47^.

In situations where bioconvective flow of carbon nanotubes is affected by Soret and Dufour reactions, influence of thermal radiation can be considerable. Therefore, thermal radiation plays a significant role in heat transfer. Nasir^48^ investigated entropy of MHD flow in porous medium under thermal radiation impacts. Dawar^49^ examined stagnation point flow with solar radiation effect. Dawar^50^ discussed nonlinear thermal radiation impact on convectively heated water based nanofluids. Bilal^51,52^ examined micro-polar fluid over porous medium with radiation regime and explored Dufour impacts on Non Newtonian fluid over stretching cylinder.

The above conducted literature review suggests that many researcher have discussed the significance of the hybrid and ternary hybrid nanofluids in the heat transfer mechanisms^1–5,27,28^. In addition to significance of their high thermal conductivity the major concern is selecting the nanoparticle which boost thermal conductivity more effectively. Keeping in view this matter, researcher have addressed this issue using the carbon nanotubes^6–10^. Noticing these highly concerning issues in heat transfer, higher thermal conductivity and suitable particle selection, in this article novelty of the work is to investigate the comparative dynamics of the bioconvective carbon nanotubes flow and heat transfer over a convectively heated stretched surface. This study undertake basically the two combinations of hybrid nanofluids one using single layer wall carbon nanotubes and second using multi wall carbon nanotubes with different working fluids. The working fluid taken into account in are ethylene glycol and mixture based working fluid known as “ethylene glycol-water”. The comparative flow dynamics are investigated for the hybrid nanofluids namely; ${\text{SWCNT}} - {\text{MWCNT}}/{\text{EG and SWCNT}} - {\text{MWCNT}}/{\text{EG}} - {\text{H}}_{2} {\text{O}}$. In our study, we also incorporate thermal radiation effect on the comparative flow dynamics in the presence of convective boundary condition. The micro-organisms upward acceleration cause bioconvection phenomenon^36–40^, we employ the motile microorganisms migration in our study to further investigate the bioconvective flow attributes. It can be noted that due to high non-linearity in the in convective term in NS momentum it is quite impossible to produce analytical solution of formulated problem. Thus, researchers use numerical methods to obtain the solutions of problems. Hayat and Nadeem^1^ used bvp-4c method, Bilal^15^ employed Parametric computation approach (PCA), Reddy and Rao^31^ examined their problem using FEM, Shah^34^ investigated Soret and Dufour effects using Optimal Homotopy analysis method, Mahdy^40^ analyzed gyrotactic microorganism using Rung-Kutta method, in this study we used the bvp-4c technique to obtain graphical and numeric outcome for comparative dynamics of the hybrid nanofluids. In this article taking into consideration the above literature review following research question will be addressed:

How does Soret and Dufour affects comparative dynamics of the bioconvective flow of the carbon nanotubes?

What is the impact of Soret and Dufour on comparative heat transfer features of hybrid nanofluids in the presence of thermal radiation?

How does rising the levels of thermal radiation, heat source/sink (Space and temperature dependent), Dufour will effect the temperature profile of hybrid nanofluids in the event of heated surface?

What is impact of inclined magnetization, mixed convection and inclination angle on comparative motion dynamics of hybrid nanofluids?

What is the impact of Soret, Dufour, mixed convection, magnetization, thermal radiation and thermal Biot number on the heat transfer and drag coefficient?

## Mathematical formulation

**Figure 1:**
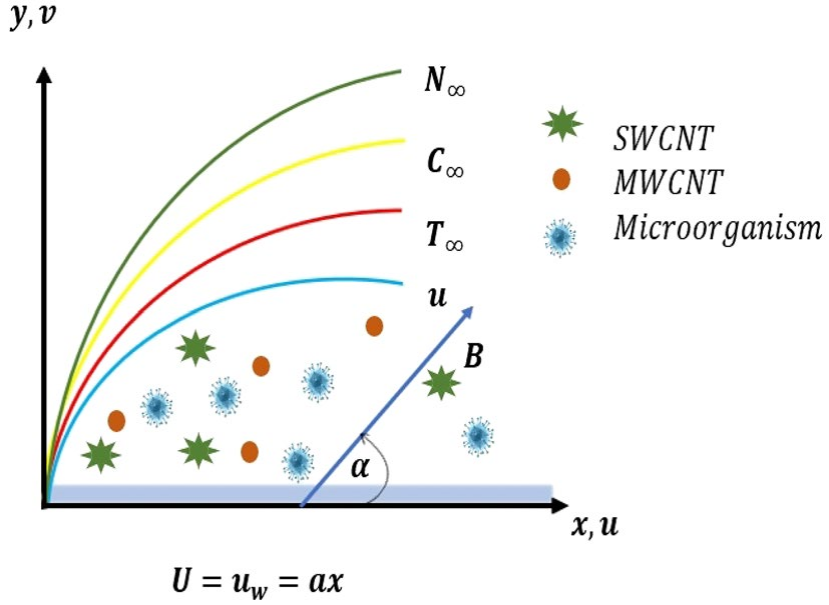
Problem configuration and coordinates system.

Consider the steady, two-dimensional, $${\text{SWCNT}} - {\text{MWCNT}}/{\text{EG and SWCNT}} - {\text{MWCNT}}/{\text{EG}} - {\text{H}}_{2} {\text{O}}$$ Prandtl hybrid nanofluids flow under the effect of inclined magnetic field over heated stretching surfaces that contains motile microorganisms in the presence of thermal radiation. The fluid flow is also taken into account at $$ y > 0$$. When an inclining magnetic field is applied to an ethylene glycol–water base hybrid nanofluid flow, an angle is produced along *x*-axis. The velocity $$u_{w} \left( x \right) $$ caused the plate to stretch along the *x*-axis. Problem configuration has been provided in the Fig. 1 to illustrate the configuration of the coordinate system. Flow governing equations, conservation of mass, conservation of momentum, conservation of energy, concentration and microorganisms for Prandtl hybrid nanofluid are given below^4,5,38^.

**Equation of continuity**

1 $$ \frac{\partial u}{{\partial x}} + \frac{\partial v}{{\partial y}} = 0. $$

**Equation of momentum**

2 $$ \begin{gathered} u\frac{\partial u}{{\partial x}} + v\frac{\partial u}{{\partial y}} = V_{hnf} \frac{A}{C}\left( {\frac{{\partial^{2} u}}{{\partial y^{2} }}} \right) + V_{hnf} \frac{A}{{2C^{3} }}\left( {\frac{{\partial^{2} u}}{{\partial^{2} y}}} \right)\left( {\frac{\partial u}{{\partial y}}} \right)^{2} \hfill \\ \;\;\; + \frac{{\sigma_{hnf} }}{{\rho_{hnf} }}B_{ \circ }^{2} u\sin^{2} \left( \alpha \right) + \frac{1}{{\rho_{hnf} }}\left[ {g\left( {T - T_{\infty } } \right)\rho_{f} \beta \left( {1 - C_{\infty } } \right)} \right. \hfill \\ \left. {\;\;\; + g\left( {C_{\infty } - C} \right)\left( {\rho_{p} - \rho_{f} } \right) - \left( {\rho_{m} - \rho_{p} } \right)\left( {N_{\infty } - N} \right)g\gamma^{\prime}} \right]. \hfill \\ \end{gathered} $$

**Equation of energy**

3 $$ \begin{aligned} v\frac{\partial T}{{\partial y}} + u\frac{\partial T}{{\partial x}} & = \alpha_{hnf} \left( {\frac{{\partial^{2} T}}{{\partial y^{2} }}} \right) - \frac{1}{{(\rho C_{p} )_{hnf} }}\frac{\partial }{\partial y}\{ q_{r} \} + \frac{{\mu_{hnf} }}{{(\rho C_{p} )_{hnf} }}\left( {\frac{\partial u}{{\partial y}}} \right)^{2} \\ & \;\;\; + \frac{1}{{(\rho C_{p} )_{hnf} }}\left( {q^{\prime\prime\prime}} \right) + \frac{{D_{2} }}{{(\rho C_{p} )_{hnf} }}\frac{{\partial^{2} C}}{{\partial y^{2} }}. \\ \end{aligned} $$

**Equation of concentration**

4 $$ v\frac{\partial C}{{\partial y}} + u\frac{\partial C}{{\partial x}} = D_{B} \frac{{\partial^{2} C}}{{\partial y^{2} }} + D_{1} \left( {\frac{{\partial^{2} T}}{{\partial y^{2} }}} \right). $$

**Equation of motile microorganism**

5 $$ v\frac{\partial N}{{\partial y}} + u\frac{\partial N}{{\partial x}} = D_{m} \frac{{\partial^{2} N}}{{\partial y^{2} }} + \frac{{bw_{c} }}{{\left( {C_{\infty } - C_{w} } \right)}} \frac{\partial }{\partial y} \left( {N\frac{\partial C}{{\partial y}}} \right). $$

Suitable boundaries are as follows^38^:

6 $$ as y = 0, u = u_{w} = ax, v = 0, \frac{\partial T }{{\partial y}} = - \left( {Tw {-} T} \right)\frac{h}{{ k_{hnf} }}, C = C_{w} , N = N_{w} , $$

7 $$ as y \to \infty , u \to 0,T \to T_{\infty } , C \to C_{\infty } , N \to N_{\infty } . $$

### Similarity transforms and dimensionless flow equations

Similarity analysis has been conducted using the following suitable similarity transform^4^.

8 $$ \eta = \sqrt {\frac{a}{{v_{f} }} } y, \Psi \left( \eta \right) = f\left( \eta \right)\sqrt {av_{f} } x , \theta \left( \eta \right) = \frac{{T - T_{\infty } }}{{T_{w} - T_{\infty } }}, \varphi \left( \eta \right) = \frac{{C - C_{\infty } }}{{C_{w} - C_{\infty } }},X\left( \eta \right) = \frac{{N - N_{\infty } }}{{N_{w} - N_{\infty } }}, $$

9 $$ u = \frac{\partial \Psi }{{\partial y}}, v = - \frac{\partial \Psi }{{\partial x}}. $$

Motsumi and Makinde^53^ used radiative heat flux $q_{r}$, the radiative heat flux has the following form:

10 $$ q_{r} = - \frac{4\sigma }{{3k^{*} }} \frac{{\partial T^{4} }}{\partial y}. $$

Here, Taylor expansion has been used to approximate the radiative heat flux and expansion has been truncated up to first order leading order term. It has been assumed that temperature difference is very small, giving us $T^{4} \cong 4T^{3}_{\infty } T - 3T^{4}_{\infty }$. Using this simplification, energy Eq. (3) is now simplified to Eq. (10a).

10a $$ \begin{gathered} v\frac{\partial T}{{\partial y}} + u\frac{\partial T}{{\partial x}} = \alpha_{hnf} \left( {\frac{{\partial^{2} T}}{{\partial y^{2} }}} \right) - \frac{1}{{(\rho C_{p} )_{hnf} }}\frac{\partial }{\partial y}\left\{ { - \frac{{4\sigma T^{3}_{\infty } }}{{3k^{*} }}\frac{4\partial T}{{\partial y}}} \right\} + \frac{{\mu_{hnf} }}{{(\rho C_{p} )_{hnf} }}\left( {\frac{\partial u}{{\partial y}}} \right)^{2} \hfill \\ \;\;\; + \frac{1}{{(\rho C_{p} )_{hnf} }}\left( {q^{\prime\prime\prime}} \right) + \frac{{D_{2} }}{{(\rho C_{p} )_{hnf} }}\frac{{\partial^{2} C}}{{\partial y^{2} }}. \hfill \\ \end{gathered} $$

Equations (2–5 and 10a) are converted into dimensionless form along with boundary conditions using above similarity transform as follows:

11 $$ C_{1} \left( {ff^{\prime\prime} - f^{\prime 2} } \right) + f^{\prime\prime\prime} \left( {\alpha_{1} + \alpha_{2} f^{\prime \prime 2} } \right) - C_{2} \left( {f^{\prime}M_{1} \sin^{\prime 2} \alpha } \right) + C_{2} \left( {\theta - Nr\varphi - NcX} \right)\lambda = 0, $$

12 $$ \frac{1}{{C_{3} }}\left[ {C_{4} + \frac{4}{3} Rd} \right]\theta^{\prime\prime} + Prf\theta^{\prime} + \frac{1}{{C_{3} }}\left( {A_{1} f^{\prime} + B_{1} \theta + Du\varphi^{\prime\prime}} \right) = 0, $$

13 $$ \varphi^{\prime\prime} + Lef\varphi^{\prime} + Sr\theta^{\prime\prime} = 0, $$

14 $$ X^{\prime\prime} - \left( {X^{\prime}\varphi^{\prime} + \left( {\omega + X} \right) \varphi^{\prime\prime}} \right)Pe + fLbX^{\prime} = 0. $$

So that Eqs. (6) and (7) are formed as

15 $$ at\; y = 0,\; f = 0, f^{\prime} = 1, \;\theta^{\prime} = - \frac{{k_{f} }}{{k_{hnf} }}Bi\left( {1 - \theta } \right),\;\varphi = 1, \;X = 1, $$

16 $$ as y \to \infty , f^{\prime} \to 0, \theta \to 0, \varphi \to 0, X \to 0 . $$

In above equations, Prandtl number is expressed as $Pr = \frac{{v_{f} \left( {\rho C_{p} } \right)_{f} }}{{k_{f} }}$, Peclet number is represented as $Pe = \frac{{bw_{c} }}{{D_{m} }}$, Dufour number is given as $Du = \frac{{D_{2} }}{{\alpha \left( {\rho C_{p} } \right)_{f} }}\frac{{\left( {C_{w} - C_{\infty } } \right)}}{{\left( {T_{w} - T_{\infty } } \right)}},$ $Le = \frac{{v_{f} }}{{D_{B} }}$ represents the Lewis number,$Nc = \frac{{\gamma^{\prime}\left( {\rho_{m} - \rho_{f} } \right)\left( {N_{\infty } - N_{w} } \right)}}{{\left( {C_{\infty } - 1} \right)\rho_{f} \beta \left( {T_{w} - T_{\infty } } \right)}}$ denotes Rayleigh number bioconvection, bioconvection constant is denoted as $\omega = \frac{{N_{\infty } }}{{N_{w} - N_{\infty } }}$,$Sr = \frac{{D_{1} }}{{v_{f} }}\frac{{\left( {T_{w} - T_{\infty } } \right)}}{{\left( {C_{w} - C_{\infty } } \right)}}$ is Soret number, $Rd = \frac{{4\sigma T_{\infty } }}{{3k^{*} k_{f} }}$ is radiation parameter, $Bi = \frac{h}{{k_{f} }}\sqrt {\frac{{v_{f} }}{a}}$ is thermal Biot number, Bioconvection Lewis number is $Lb = \frac{{v_{f} }}{{D_{m} }}$, mixed convection parameter shows as $\lambda = \frac{{\left( {1 - C_{\infty } } \right)\beta g\left( {T_{w} - T_{\infty } } \right)}}{{ax^{2} }}$, $\alpha_{1} { } = \frac{{\text{A}}}{{\text{C}}}$ depicts Prandtl fluid parameter, $M = \frac{{\sigma_{f} B_{^\circ }^{2} }}{{\rho_{f} a}}$ indicate magnetization force,$A_{1} = \frac{A}{{a\left( {\rho C_{p} } \right)_{f} }}$ is Space dependent heat source/sink, $\alpha_{2} = \frac{{a^{3} x^{2} A}}{{2v_{f} C^{3} }}$ is known as elastic parameter,$B_{1} = \frac{B}{{a\left( {\rho C_{p} } \right)_{f} }}$ is temperature dependent heat source/sink, $Nr = \frac{{\left( {\rho_{p} - \rho_{f} } \right)\left( {C_{w} - C_{\infty } } \right)}}{{(1 - C_{\infty } )\rho_{f} \beta \left( {T_{w} - T_{\infty } } \right)}}$ denotes the buoyancy ratio parameter. Relations (17–20) are modified thermophysical relation employed in this study.

17 $$ C_{1} = \left( {1 - \varphi_{1} } \right) + \varphi_{1} \frac{{\rho_{{s_{1} }} }}{{\rho_{s} }}\left( {1 - \varphi_{2} } \right) + \varphi_{2} \frac{{\rho_{{s_{2} }} }}{{\rho_{s} }}(1 - \varphi_{2} )^{2.5} (1 - \varphi_{1} )^{2.5} , $$

18 $$ C_{2} = (1 - \varphi_{2} )^{2.5} (1 - \varphi_{1} )^{2.5} , $$

19 $$ C_{3} = \left( {\left( {1 - \varphi_{1} } \right) + \varphi_{1} \frac{{\left( {\rho C_{P} } \right)_{{S_{1} }} }}{{\left( {\rho C_{P} } \right)_{f} }}} \right)\left( {1 - \varphi_{2} } \right) + \frac{{\left( {\rho C_{P} } \right)_{{S_{1} }} }}{{\left( {\rho C_{P} } \right)_{f} }} \varphi_{1 } , $$

20 $$ C_{4} = \frac{{\left( {k_{{b_{f} - k_{{s_{2} }} }} } \right)\varphi_{2} + k_{{s_{2} }} + \left( {m - 1} \right)k_{{b_{f} }} }}{{\left( {m - 1} \right)k_{f} + k_{{s_{2} }} - \left( {m - 1} \right)\left( {k_{f} - k_{{s_{2} }} } \right)\varphi_{2} }}. \frac{{\left( {k_{f} - k_{{s_{1} }} } \right)\varphi_{1} + k_{{s_{1} }} + \left( {m - 1} \right)k_{f} }}{{\left( {m - 1} \right)k_{f} + k_{{s_{1} }} - \left( {m - 1} \right)\left( {k_{f} - k_{{s_{1} }} } \right)\varphi_{1} }}. $$

The physical quantities can be calculated as^43^:

21 $$ C_{fx} = \frac{{\tau_{w} }}{{\rho_{f} u_{w}^{2} }}\; and\; {\rm N}^{*} u_{x} = \frac{{xq^{*}_{w} }}{{k_{f} \left( {T_{w} - T_{\infty } } \right)}}{ }. $$

22 $$ q^{*}_{w} = - k_{hnf} \left( {\frac{\partial T}{{\partial y}}} \right)_{y = 0} and \tau_{w} = \mu_{hnf} \left( {\frac{A}{C}\frac{\partial u}{{\partial y}} + \frac{A}{{2C^{3} }}\left( {\frac{\partial u}{{\partial y}}} \right)^{3} } \right). $$

Dimensionless form of these physical quantities are given as below:

23 $$ C_{fx} = \frac{1}{{C_{2} }}R_{{e_{x} }}^{ - 0.5} \left( {\alpha_{1} + \alpha_{1} f^{\prime\prime}(0)^{2} } \right)f^{\prime\prime}\left( 0 \right), $$

24 $$ {\rm N}^{*} u_{x} = - \frac{{k_{hnf} }}{{k_{f} }} R_{{e_{x} }}^{0.5} \theta^{\prime}\left( 0 \right). $$

So, relevant Reynolds number is $R_{{e_{x} }} = \frac{{{\varvec{u}}_{w} x}}{{v_{f} }}$. Now the useful thermo-physical properties of nanoparticles and Ethylene glycol–water are as follows^40,41^, and shown in Table 1. Also thermo-physical properties are given as follows^42^, are given in Table 2.

**Table 1:** Thermophysical properties of MWCNT, ethylene glycol, SWCNT and ethylene glycol–water^40,41^.

NP & basefluids	$\rho \left( {kg m^{ - 3} } \right)$	$C_{p} \left( {Jkg^{ - 1} K^{ - 1} } \right)$	$k\left( {Wm^{ - 1} K^{ - 1} } \right)$	$Pr$
${\text{SWCNT}}$	2600	425	6600	
${\text{MWCNT}}$	1600	796	3000	
${\text{Ethylene glycol}}$	1109	2400	0.258	23.50
${\text{H}}_{2} {\text{O}}$	1063.8	3630	0.387	7.2

**Table 2:** Hybrid nanofluid’s thermophysical properties^42^.

Thermo physical properties	Hybrid nanofluid
Thermal conductivity	$\frac{{k_{hnf} }}{{k_{bf} }} = \frac{{\left( {k_{{b_{f} - k_{{s_{2} }} }} } \right)\varphi_{2} + k_{{s_{2} }} + \left( {m - 1} \right)k_{{b_{f} }} }}{{\left( {m - 1} \right)k_{f} + k_{{s_{2} }} - \left( {m - 1} \right)\left( {k_{f} - k_{{s_{2} }} } \right)\varphi_{2} }},$
	Where $\frac{{k_{bf} }}{{k_{f} }} = \frac{{\left( {k_{f} - k_{{s_{1} }} } \right)\varphi_{1} + k_{{s_{1} }} + \left( {m - 1} \right)k_{f} }}{{\left( {m - 1} \right)k_{f} + k_{{s_{1} }} - \left( {m - 1} \right)\left( {k_{f} - k_{{s_{1} }} } \right)\varphi_{1} }} .$
Density	$\rho_{hnf} = \left( {1 - \varphi_{2} } \right) + \varphi_{1} \frac{{\rho_{{s_{1} }} }}{{\rho_{f} }}\left( {1 - \varphi_{1} } \right)\rho_{f} + \varphi_{2} \rho_{{s_{2} }} .$
Dynamic viscosity	$\mu_{hnf} = \frac{{\mu_{f} }}{{(1 - \varphi_{2} )^{2.5} (1 - \varphi_{1} )^{2.5} }}.$
Heat capacity	$\left( {\rho C_{P} } \right)_{hnf} = \left( {\rho C_{P} } \right)_{f} \left( {1 - \varphi_{2} } \right)\left( {\left( {1 - \varphi_{1} } \right) + \varphi_{1} \frac{{\left( {\rho C_{P} } \right)_{{S_{1} }} }}{{\left( {\rho C_{P} } \right)_{f} }}} \right).$

## Numerical solution

**Figure 2:**
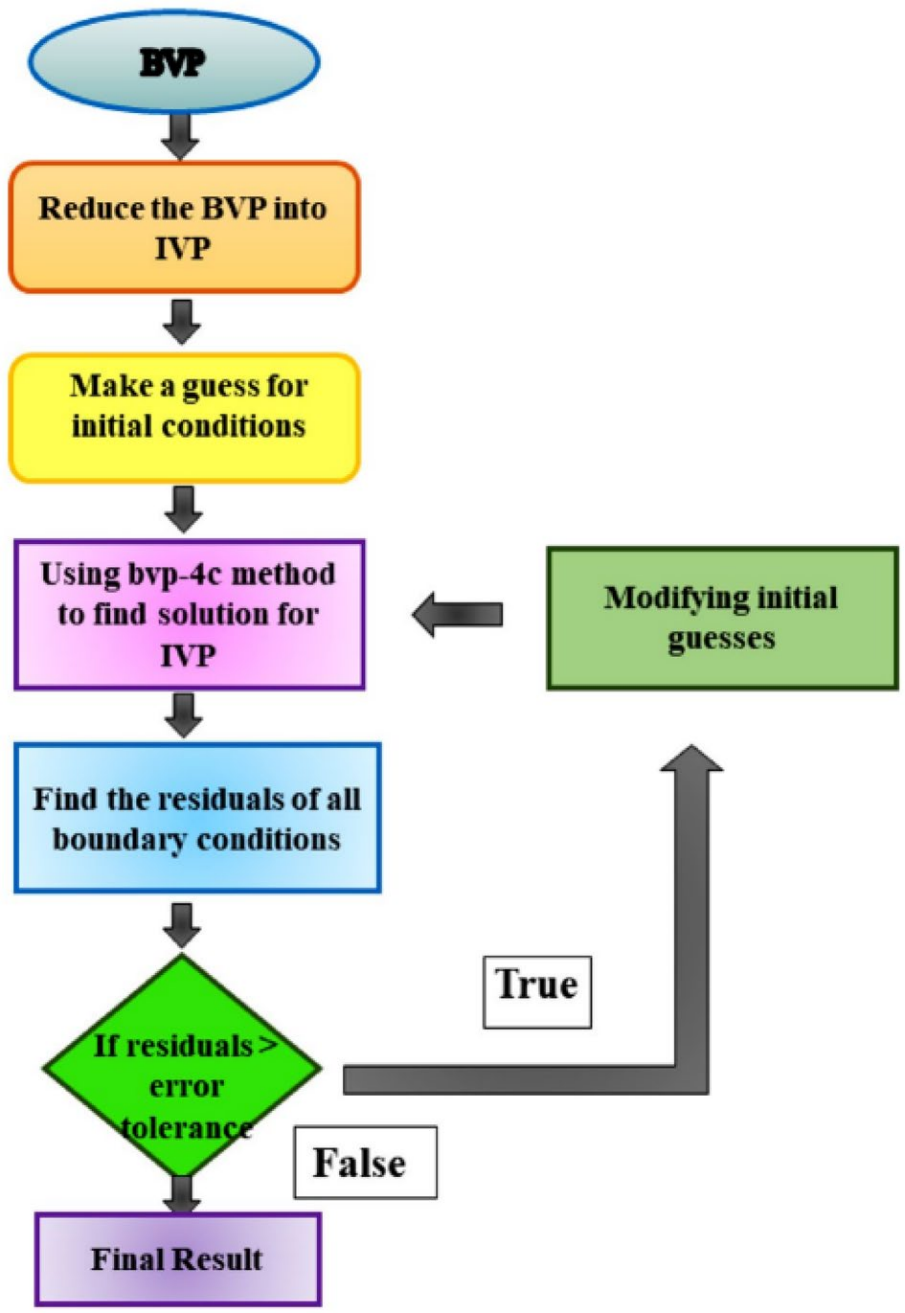
Chart of numerical method steps.

Hayat and Nadeem^1^ used bvp-4c method, Bilal^15^ employed Parametric computation approach (PCA), Reddy and Rao^31^ examined their problem using FEM, Shah^34^ investigated Soret and Dufour effects using Optimal Homotopy analysis method, Mahdy^40^ analyzed gyrotactic microorganism using Rung-Kutta method, in this study we used the bvp-4c technique to obtain graphical and numeric outcome for comparative dynamics of the hybrid nanofluids. There are certain benefits of this computational technique which were taken into consideration while selecting a technique to solve present problem. Following are the key efficient indicators of bvp-4c: bvp-4c technique is more simple to implement, low computational cost, time saving and highly accurate and easy to understand. The whole solution algorithm is displayed in Fig. 2.

The dimensionless Eqs. (11–14) with boundary conditions (15, 16) are tackled numerically with MATLAB. The new set of variables is given in the Eq. (25) and transformed flow governing equations and boundaries are given in the (26–29) and (30, 31), respectively. These newly formulated equations are then tackled with bvp-4c technique in MATLAB.

25 $$ \begin{aligned} f^{\prime} & = S_{2} , f = S_{1} , f^{\prime\prime} = S_{3} , f^{\prime\prime\prime} = SS_{1} , \theta = S_{4} , \theta^{\prime} = S_{5} , \theta^{\prime\prime} = SS_{1} , \\ \varphi & = S_{6} , \varphi^{\prime} = S_{7} , \varphi^{\prime\prime} = SS_{3} , \chi = S_{8} , \chi^{\prime} = S_{9} , \chi^{\prime\prime} = SS_{4} . \\ \end{aligned} $$

26 $$ ss_{1} = \frac{1}{{\left( {\alpha_{1} + \alpha_{2} s_{3} } \right)}}\left( {\left( {C_{2} s_{2} M \sin \alpha^{2} } \right) - C_{1} \left( {s_{1} s_{3} - s^{2}_{2} } \right) - C_{2} \lambda \left( {s_{4} - Nrs_{5} - Ncs_{8} } \right)} \right), $$

27 $$ ss_{2} = \frac{{ - Pr\left( {s_{1} s_{5} } \right) - \frac{1}{{C_{3} }}\left( {A_{1} S_{2} + B_{1} S_{4} + DuSS_{3} } \right)}}{{\frac{1}{{C_{3} }}\left[ {C_{4} + \frac{4}{3} Rd} \right]}}, $$

28 $$ ss_{3} = - Les_{1} s_{7} - Srss_{2} , $$

29 $$ ss_{4} = \left( {s_{8} s_{9} + \left( {\omega + s_{8} } \right)ss_{3} } \right)Pe - Lbs_{1} s_{9} . $$

Transformed boundary conditions:

30 $$ {\text{When }}\eta \to 0 , s_{2} = 1, s_{1} = 0, s_{8} = 1, s_{5} = \frac{{k_{f} }}{{k_{hnf} }}Bi\left( {\theta - 1} \right), s_{6} = 1. $$

31 $$ {\text{ When }}\eta \to \infty ,{ }s_{6} \to 0, s_{4} \to 0, s_{2} \to 0, s_{8} \to 0. $$

### Validation of outcomes

Present results are validated using bvp-4c code for the skin friction with already published results. It is assumed that when the magnetization force is varied and elasticity level is kept at unity along the inclination of magnetization is 90 degrees and all the other study parameters are taken equal to zero, the outcome of present study are in great agreement with those of Jalil^54^ and Ali^55^. This validates the numerical method used to achieve data in this study as well the outcome under certain parametric values. The performed validation has been given in the Table 3 below.

**Table 3:** Comparison of skin coefficient keeping inclination angle at 90 degrees, Prandtl fluid value at 1 with varying magnetization force and keeping all other parametric values equal to zero.

M	Jalil et al.^44^	Ali et al.^45^	Our Outcomes
0.0	1.000000	1.0000080	1.00000890
0.2	1.095445	1.0954458	1.09544490
1.0	1.414214	1.4142132	1.41421356
1.2	1.483340	1.4832393	1.48323932

## Discussion and results

In this section influence of the nondimensional numbers on the comparative dynamics of ${\text{SWCNT}} - {\text{MWCNT}}/{\text{EG and SWCNT}} - {\text{MWCNT}}/{\text{EG}} - {\text{H}}_{2} {\text{O}}$ the Prandtl hybrid nanofluids have been presented and discussed. The impact of inclined magnetization $0.5 \le {\text{M}} \le 4$, mixed convection $0.01 \le {\uplambda } \le 0.04$, angle of inclination $0 \le {\upalpha } \le 180$, thermal radiation $0.1 \le {\text{Rd}} \le 1.7$, Dufour effect $0.1 \le {\text{Du}} \le 0.19$, space dependent and temperature heat source/sink $0.1 \le {\text{A}}_{1} /{\text{B}}_{1} \le 0.9$, bioconvection Lewis number $0.5 \le {\text{Lb}} \le 0.9$, Peclet number $1 \le {\text{Pe}} \le 1.5$, Soret effect $0.1 \le {\text{Sr}} \le 0.9$ and Lewis number $0.5 \le {\text{Le}} \le 2.4$ has been discussed comparatively. The impact of these study parameters has been depicted on velocity, temperature, concentration and microorganisms profiles. Moreover, tabulated data sets have been generated for different hybrid nanofluids under examination to illustrate the behavior of skin friction and Nusselt number under the varying levels of study parameters.

### Result analysis

In this analysis outcomes of skin friction are validated under assigned values of present study parameters with those of Jalil^54^ and Ali et al.^55^. We validate the present outcomes the magnetization force has been varied over the heated surface and by keeping the angle of inclination of magnetization force at 90 degrees. Further, all the other dimensionless numbers have been assumed to be equal to zero. It must be noted that here we have taken elasticity of the Prandtl fluid equal to unity to make more rigorous comparison with the results of Jalil^54^ and Ali et al.^55^. Table 3 shows the comparison between our results and findings of other research and demonstrate a good agreement between the results.

### Velocity profile (${\mathbf{SWCNT}} - {\mathbf{MWCNT}}/{\mathbf{EG}} {\mathbf{and}} {\mathbf{SWCNT}} - {\mathbf{MWCNT}}/{\mathbf{EG}} - {\mathbf{H}}_{2} {\mathbf{O}}$)

**Figure 3:**
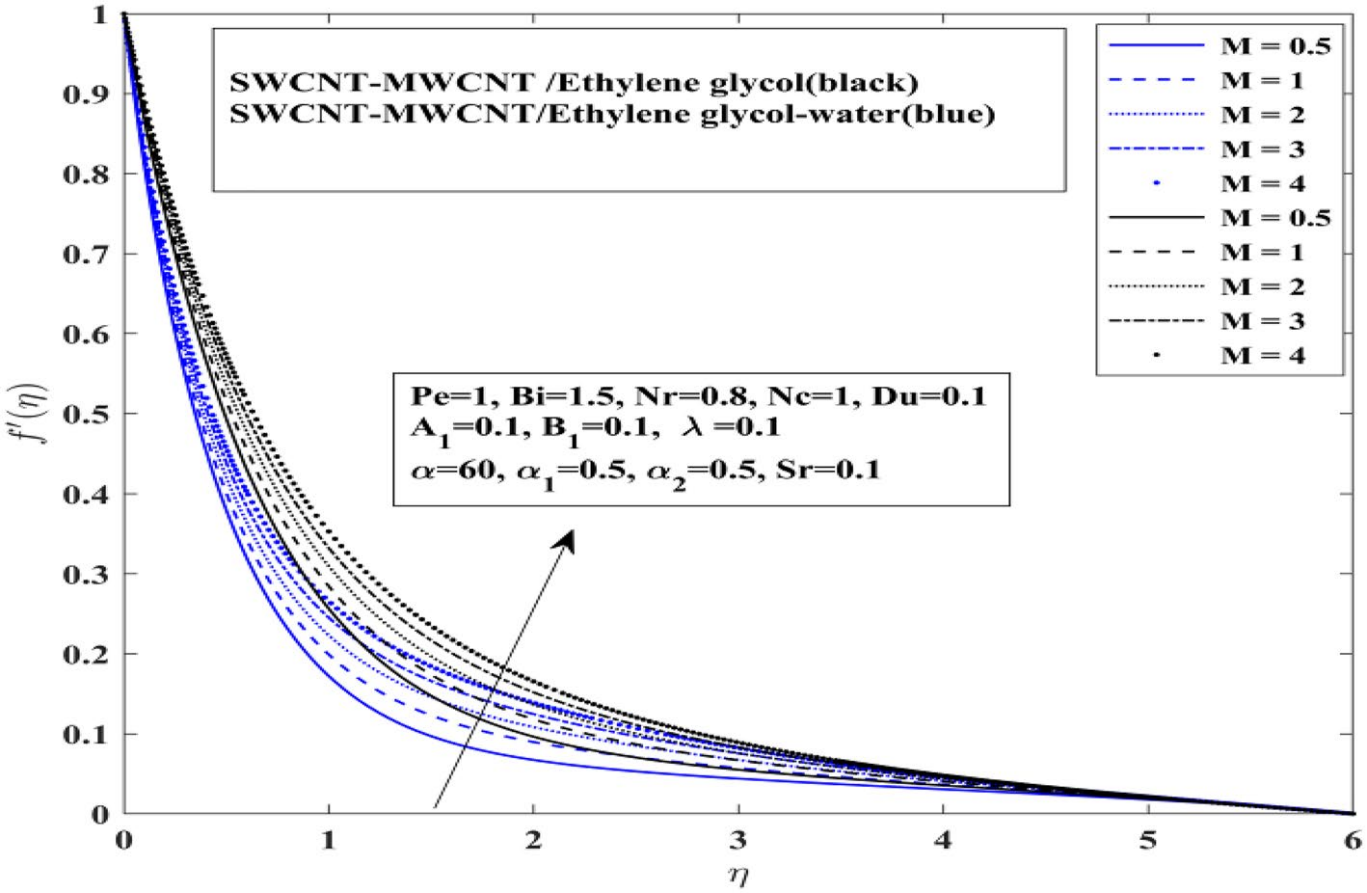
Impact of magnetization force *M* on $f^{\prime}$.

Comparative dynamics of velocity profile for hybrid nanofluids $\left( {{\text{SWCNT}} - {\text{MWCNT}}/{\text{EG and SWCNT}} - {\text{MWCNT}}/{\text{EG}} - {\text{H}}_{2} {\text{O}}} \right)$ under the effect of magnetization force, mixed convection and angle of inclination has been presented in this section. The influence of magnetization force is presented on the velocity profile in the Fig. 3. Raising levels of magnetization force or magnetic field will generate the resistive force better known as Lorentz force in the fluid and Lorentz force will consequently act as resistive force against the fluid acceleration. As the results the fluid near the heated stretched surface will accelerate away form the surface. In other words higher magnetization responsible for rapid and abrupt decline in fluid motion and it is evident from the demonstrated profile of velocity profile in Fig. 3 that under high magnetization fluid motion accelerate. It is more interesting to note here that mixture base hybrid nanofluid $\left( {{\text{SWCNT}} - {\text{MWCNT}}/{\text{EG}} - {\text{H}}_{2} {\text{O}}} \right)$ accelerates slowly in comparison with single base hybrid nanofluid $({\text{SWCNT}} - {\text{MWCNT}}/{\text{EG}}$. This result verify the fact that mixture base working fluid has higher viscosity than the single base working fluid. Additionally, higher viscosity will require more higher magnetization force to decelerate the fluid more rapidly.

**Figure 4:**
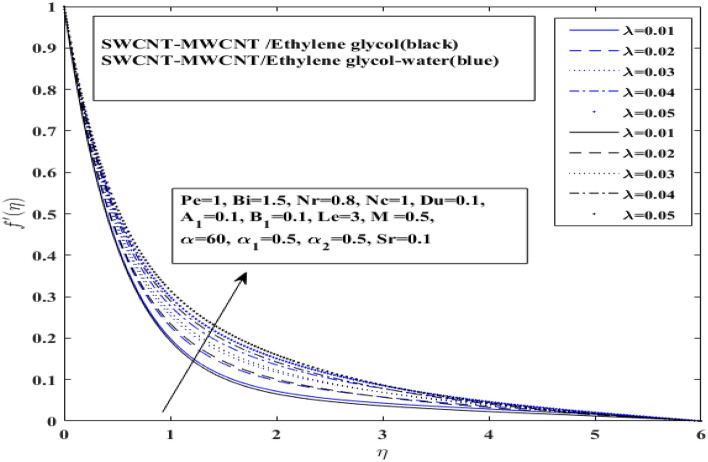
Impact of mixed convection $\lambda$ on $f^{\prime}$.

Behavior of velocity profile under effect of raising mixed convection levels for hybrid nanofluids $\left( {{\text{SWCNT}} - {\text{MWCNT}}/{\text{EG and SWCNT}} - {\text{MWCNT}}/{\text{EG}} - {\text{H}}_{2} {\text{O}}} \right)$ is depicted in Fig. 4. The velocity profile of hybrid nanofluids enhance with raising mixed convection values. It indicates that velocity of $\left( {{\text{SWCNT}} - {\text{MWCNT}}/{\text{EG}}} \right)$ hybrid nanofluid increases slower than ${ }\left( {{\text{SWCNT}} - {\text{MWCNT}}/{\text{EG}} - {\text{H}}_{2} {\text{O}}} \right)$ hybrid nanofluid. The cause of this tendency is that the fluid in outer layer is accelerated by the positive mixed convection, which functions as a favorable pressure gradient. The velocity of $\left( {{\text{SWCNT}} - {\text{MWCNT}}/{\text{EG}} - {\text{H}}_{2} {\text{O}}} \right)$ increases rapidly when compared to that of $\left( {{\text{SWCNT}} - {\text{MWCNT}}/{\text{EG}}} \right)$ hybrid nanofluid. It is worth mentioning here that there is a slight difference in increasing velocity profile for both types of hybrid nanofluids.

**Figure 5:**
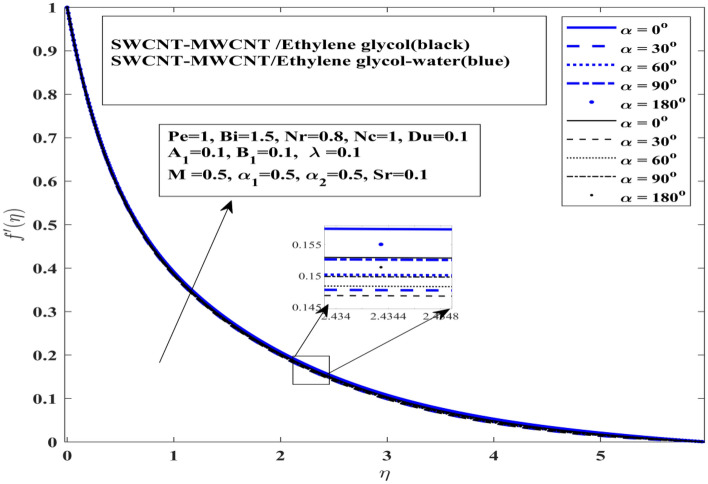
Influence inclination angle $\alpha$ on $f^{\prime}$.

Comparative outcomes of velocity profile has been presented under effect of angle of inclination of magnetization force in Fig. 5. This profile elucidates that angle of inclination impact both $\left( {{\text{SWCNT}} - {\text{MWCNT}}/{\text{EG and SWCNT}} - {\text{MWCNT}}/{\text{EG}} - {\text{H}}_{2} {\text{O}}} \right)$ hybrid nanofluids on in quite similar fashion. With increase in angle of inclination of magnetization casues the fluid to accelerate more rapidly. Moreover, it indicates that velocity of the $\left( {{\text{SWCNT}} - {\text{MWCNT}}/{\text{EG}}} \right)$ hybrid nanofluid increases slower than $\left( {{\text{SWCNT}} - {\text{MWCNT}}/{\text{EG}} - {\text{H}}_{2} {\text{O}}} \right)$ hybrid nanofluid. This also indicates that inclination angle plays significant role in achieving higher velocity profile. When the angle of inclination is small the fluid will accelerate more rapidly but the thickness of the boundary layer decrease. On the other hand when inclination angle is enhanced the boundary layer thickness contracts. The presence of strong magnetization also contributes in this regard.

### Temperature profile (${\mathbf{SWCNT}} - {\mathbf{MWCNT}}/{\mathbf{EG}} {\mathbf{and}} {\mathbf{SWCNT}} - {\mathbf{MWCNT}}/{\mathbf{EG}} - {\mathbf{H}}_{2} {\mathbf{O}}$)

**Figure 6:**
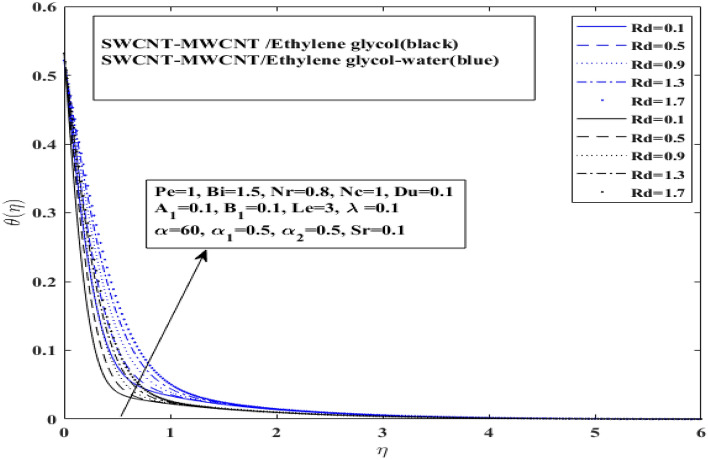
Influence of thermal radiation $Rd$ on $\theta$.

The effect of *Rd* on both $\left( {{\text{SWCNT}} - {\text{MWCNT}}/{\text{EG and SWCNT}} - {\text{MWCNT}}/{\text{EG}} - {\text{H}}_{2} {\text{O}}} \right)$ hybrid nanofluid is illustrated in Fig. 6. It is observed that with increment in thermal radiation parameter the temperature profile has increased for $\left( {{\text{SWCNT}} - {\text{MWCNT}}/{\text{EG and SWCNT}} - {\text{MWCNT}}/{\text{EG}} - {\text{H}}_{2} {\text{O}}} \right)$ hybrid nanofluid. Moreover, it indicates that the temperature curve of the $\left( {{\text{SWCNT}} - {\text{MWCNT}}/{\text{EG}}} \right)$ hybrid nanofluid increases faster than $\left( {{\text{SWCNT}} - {\text{MWCNT}}/{\text{EG}} - {\text{H}}_{2} {\text{O}}} \right)$ hybrid nanofluid. The temperature of the Prandtl hybrid nanofluid drops as the consequence of the increase in random motion, which elevates the average kinetic energy of fluid particles. This fact can be attributed to the radiation parameter is ratio of boltzmann constant to thermal conductivity of the working fluid. Thus, higher thermal conductivity corresponds to greater rate of heat transfer for the hybrid nanofluid in this case mixture base hybrid nanofluid $\left( {{\text{SWCNT}} - {\text{MWCNT}}/{\text{EG}} - {\text{H}}_{2} {\text{O}}} \right)$ as compared to mono hybrid nanofluid $\left( {{\text{SWCNT}} - {\text{MWCNT}}/{\text{EG}}} \right)$.

**Figure 7:**
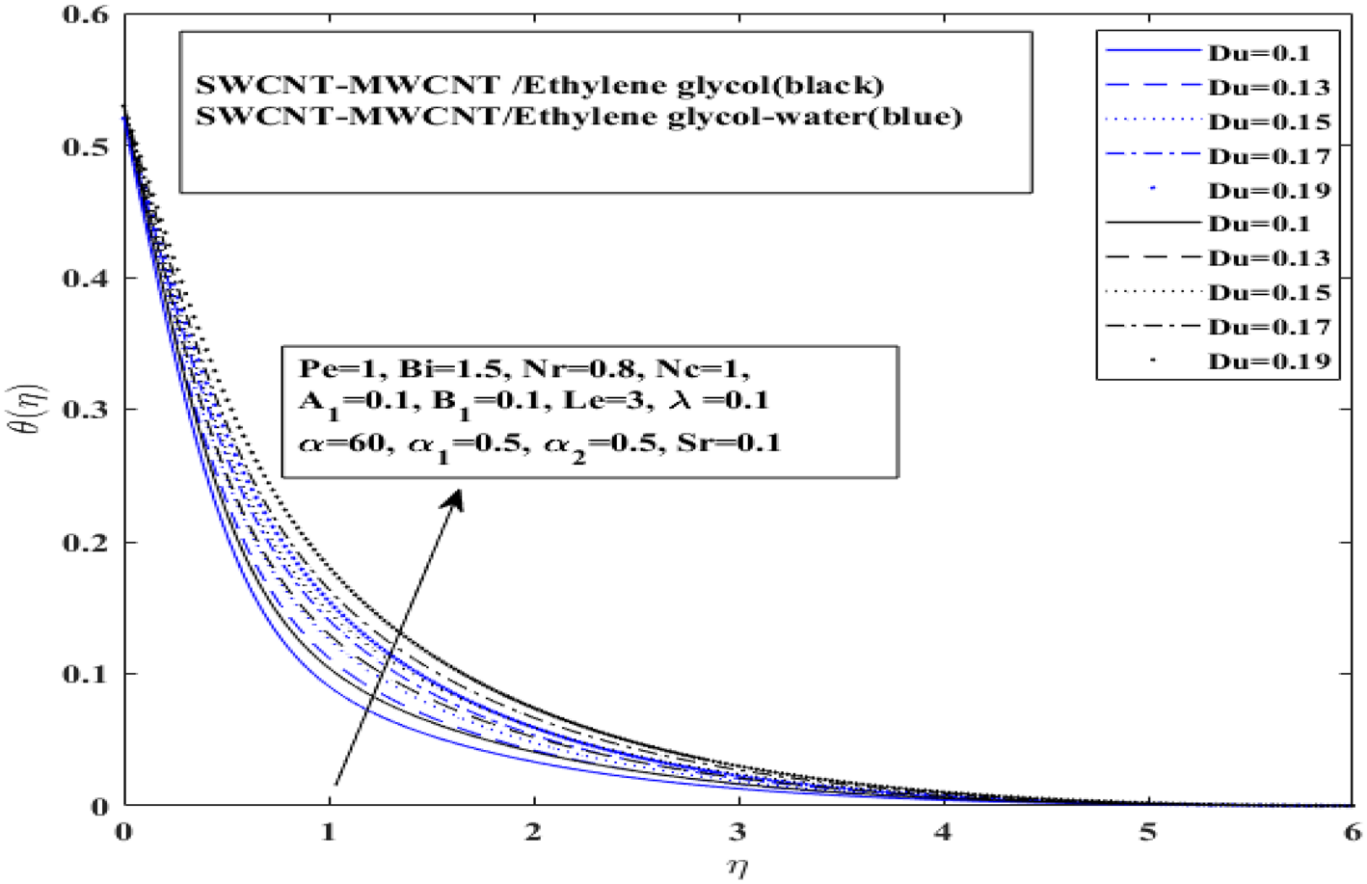
Influence of Dufour number $Du$ on $\theta$.

**Figure 8:**
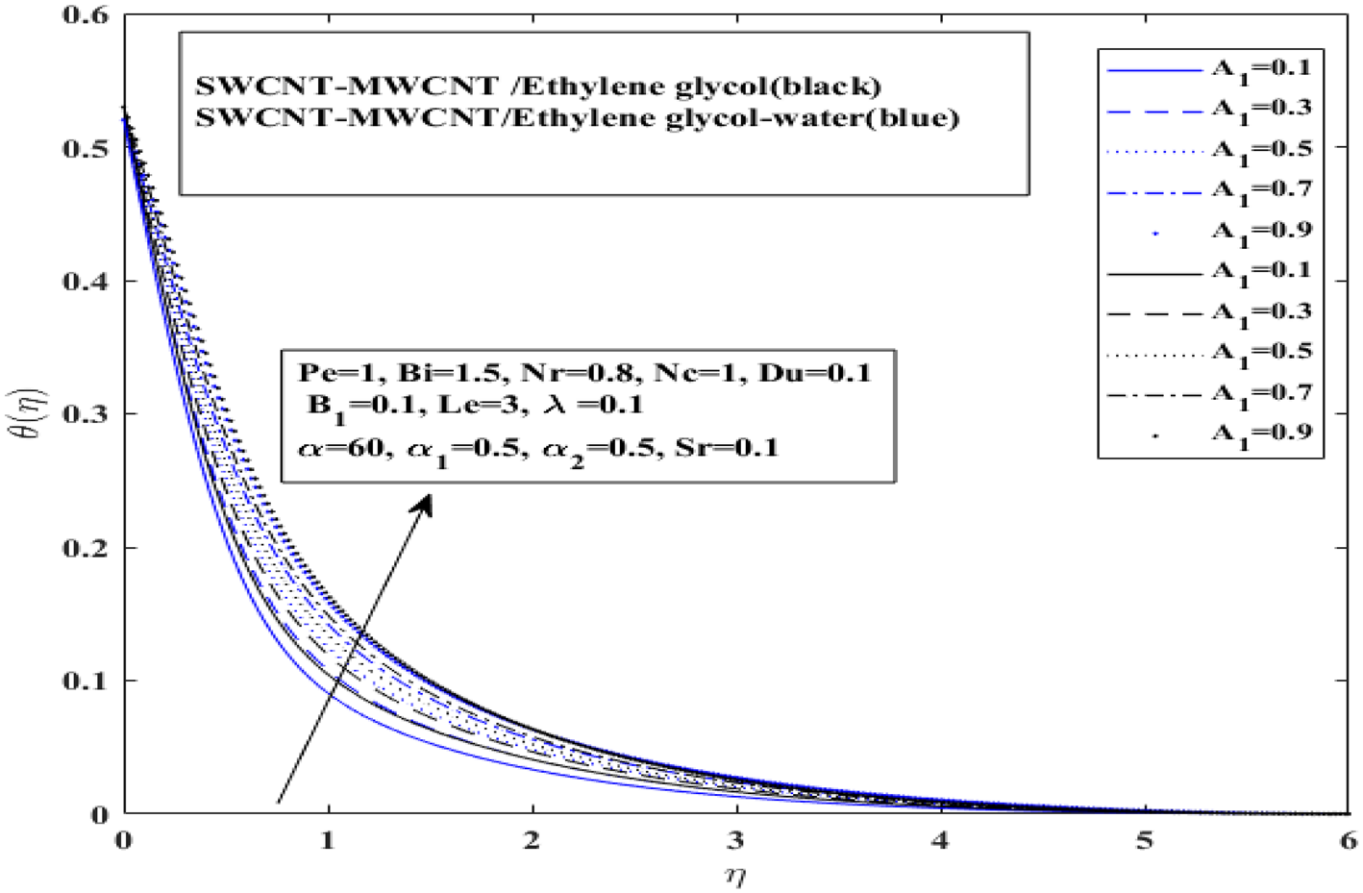
Influence of space dependent heat source of $A_{1}$ on $\theta$.

**Figure 9:**
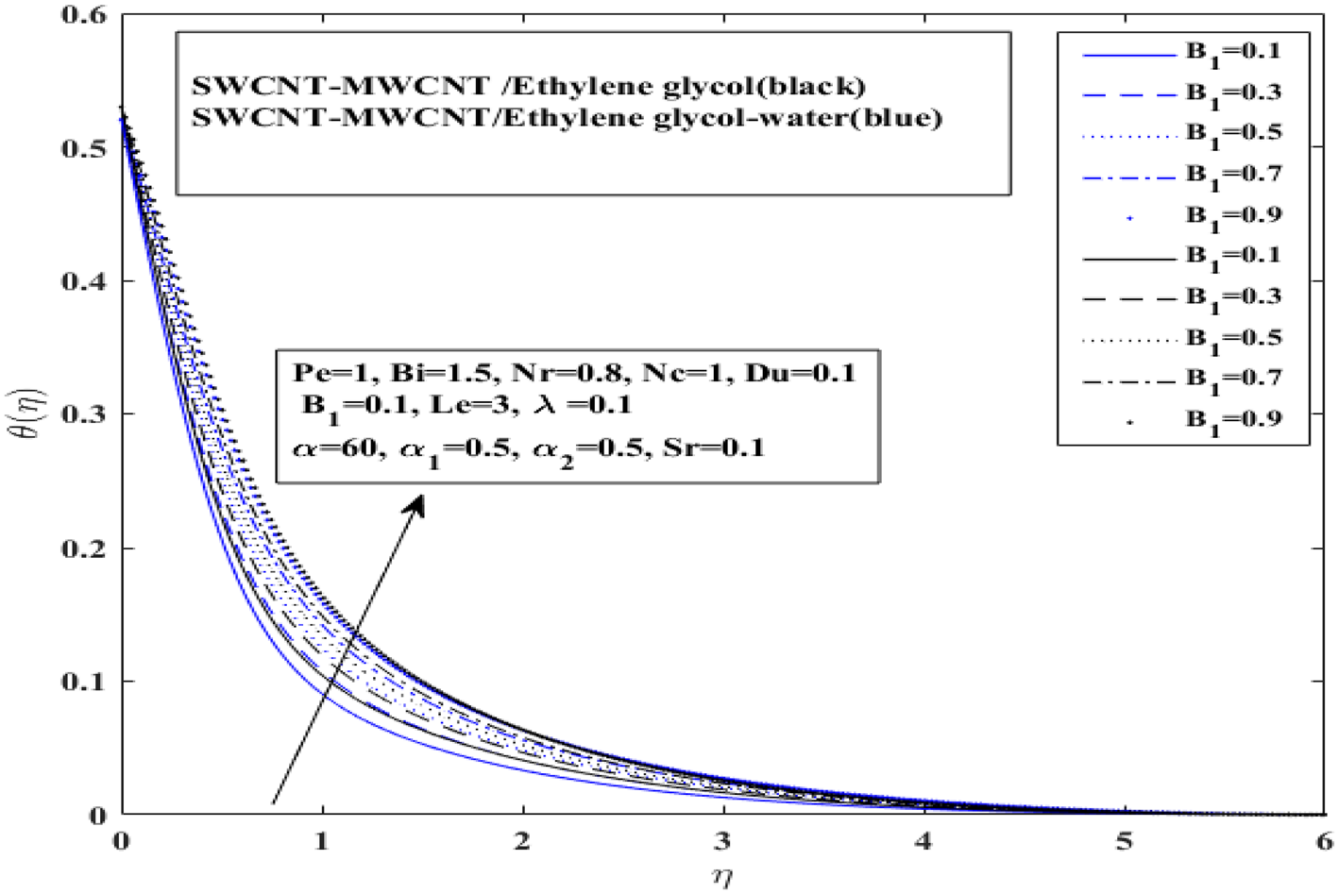
Influence of temperature dependent heat source $B_{1}$ on $\theta$.

Figures 7, 8 and 9 illustrate how Dufour $\left( {Du} \right)$, space dependent $\left( {A_{1} } \right)$ and temperature dependent $\left( {B_{1} } \right)$ heat sources affect temperature for both $\left( {{\text{SWCNT}} - {\text{MWCNT}}/{\text{EG and SWCNT}} - {\text{MWCNT}}/{\text{EG}} - {\text{H}}_{2} {\text{O}}} \right)$ hybrid nanofluids. Dufour effect on the temperature profile has been presented for both types of hybrid nanofluids $\left( {{\text{SWCNT}} - {\text{MWCNT}}/{\text{EG and SWCNT}} - {\text{MWCNT}}/{\text{EG}} - {\text{H}}_{2} {\text{O}}} \right)$ in Fig. 7. Basically, Dufour effect is the energy flux concentration gradient as illustrated by the ratio of dimensionless number. It represent the ratio of mass diffusion coefficient to thermal diffusivity. So, as the thermal diffusivity enhance the mass diffusion decrease resulting decline in Dufour effect on temperature profile. Moreover, it has been identified that temperature profile for the $\left( {{\text{SWCNT}} - {\text{MWCNT}}/{\text{EG}}} \right)$ and $\left( {{\text{SWCNT}} - {\text{MWCNT}}/{\text{EG}} - {\text{H}}_{2} {\text{O}}} \right)$ base hybrid nanofluid has risen with an increase in the Dufour number. Space dependent heat source $\left( {A_{1} } \right)$ and temperature dependent heat source $(B_{1} )$ impacts on temperature profile have been presented on in Figs. 8 and 9 for both type of hybrid nanofluids $\left( {{\text{SWCNT}} - {\text{MWCNT}}/{\text{EG and SWCNT}} - {\text{MWCNT}}/{\text{EG}} - {\text{H}}_{2} {\text{O}}} \right)$, respectively.

It has been observed that $$\left( {A_{1} } \right)$$ space dependent heat source and ($$B_{1} )$$ temperature dependent heat source both enhance temperature profile for both type of hybrid nanofluids $$\left( {{\text{SWCNT}} - {\text{MWCNT}}/{\text{EG and SWCNT}} - {\text{MWCNT}}/{\text{EG}} - {\text{H}}_{2} {\text{O}}} \right)$$ with a very slight difference. Additionally, it shows that the $$\left( {{\text{SWCNT}} - {\text{MWCNT}}/{\text{EG}}} \right){ }$$ nanofluid temperature grows more rapidly than $$\left( {{\text{SWCNT}} - {\text{MWCNT}}/{\text{EG}} - {\text{H}}_{2} {\text{O}}} \right)$$ base hybrid nanofluid temperature curve. This occurs as a result of the interplay between the response of the magnetic field and velocity of fluid particles. This contact reduces the velocity field, leading to frictional heating between liquid layers, which increases the thickness of the thermal boundary layer and improves heat transmission.

### Microorganism and concentration profiles (${\mathbf{SWCNT}} - {\mathbf{MWCNT}}/{\mathbf{EG}} {\mathbf{and}} {\mathbf{SWCNT}} - {\mathbf{MWCNT}}/{\mathbf{EG}} - {\mathbf{H}}_{2} {\mathbf{O}}$)

**Figure 10:**
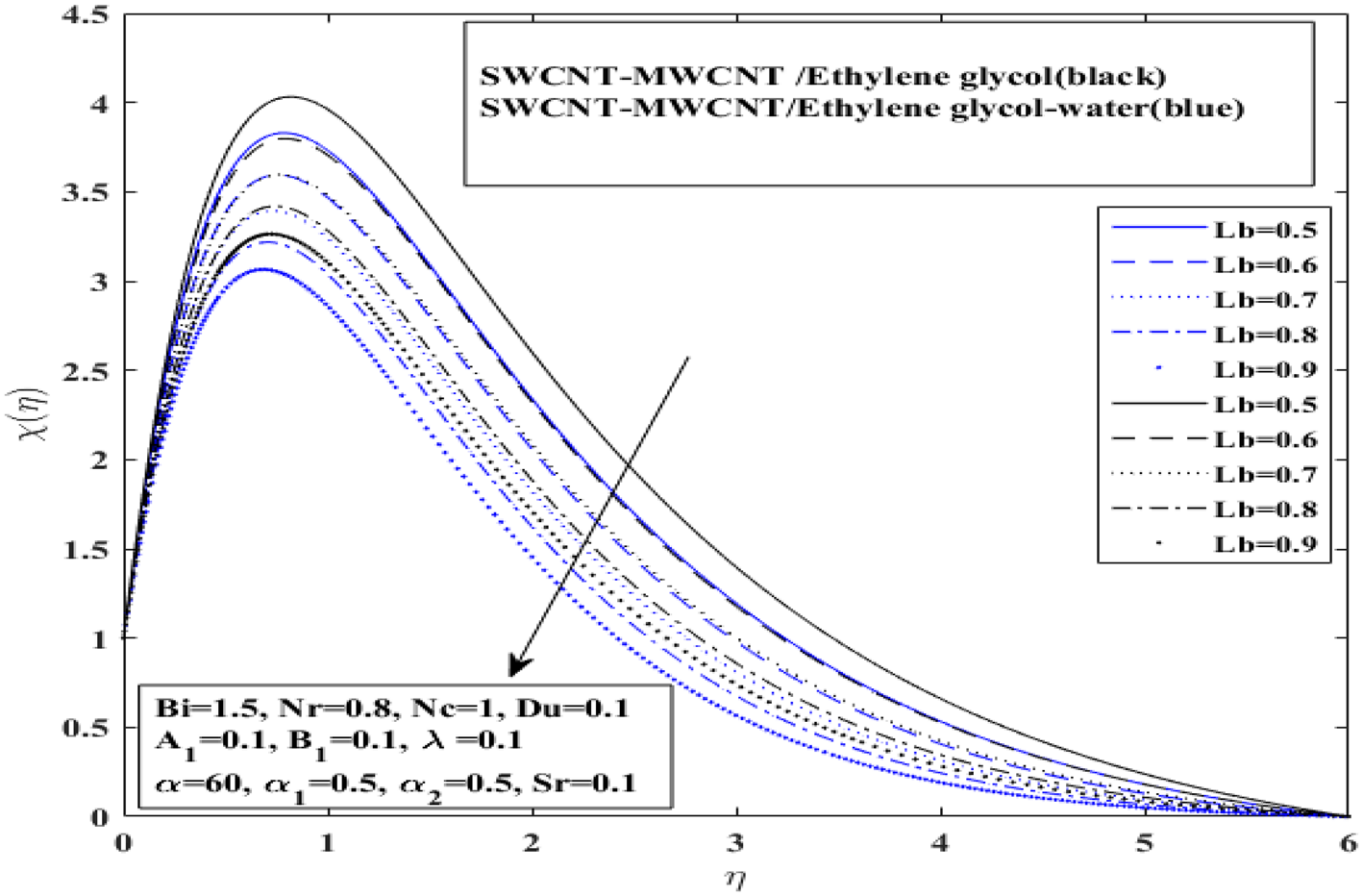
Impact of Bioconvection Lewis number *Lb* on $X$.

**Figure 11:**
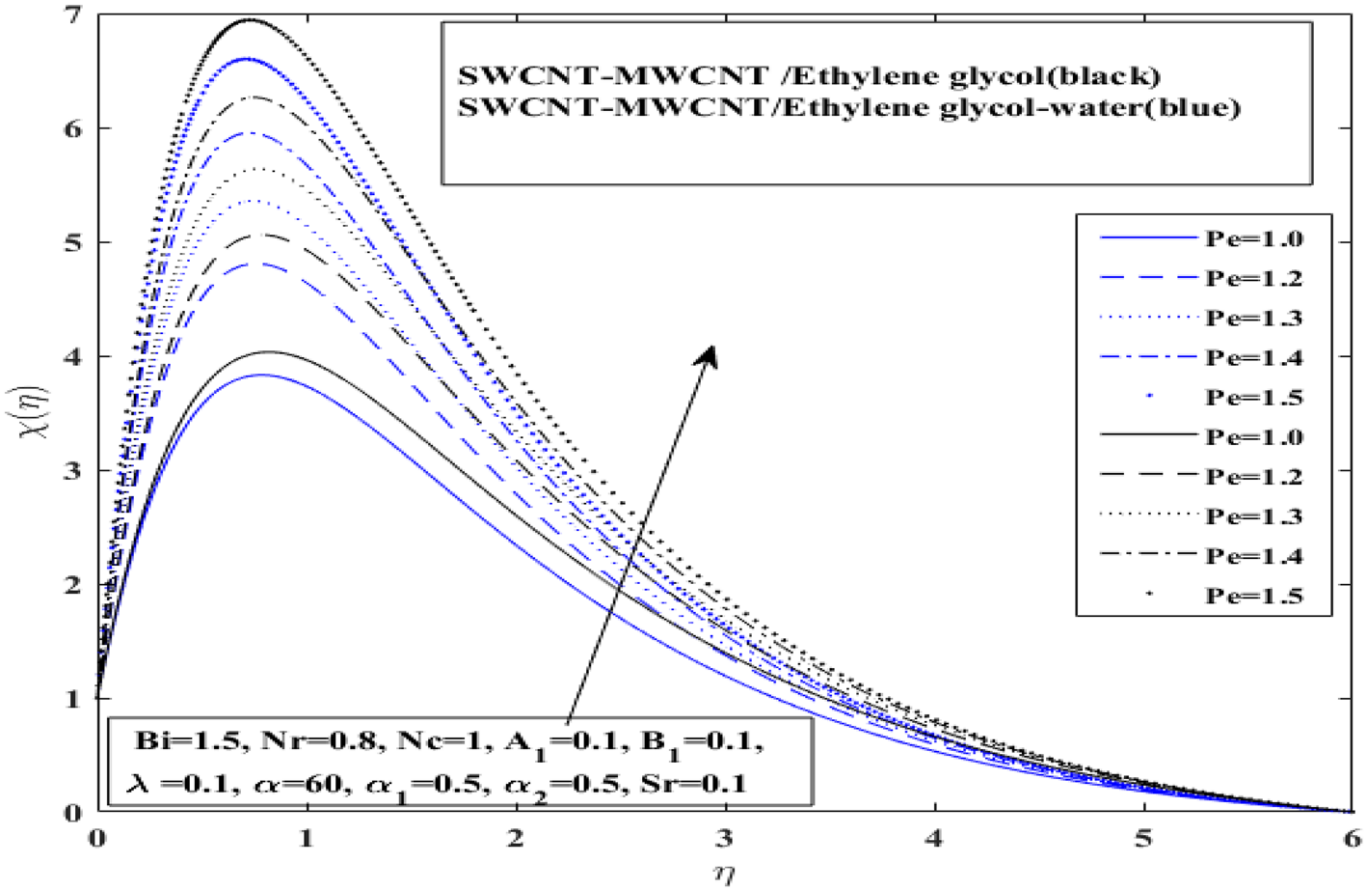
Impact of Peclet number *Pe* on $X$.

**Figure 12:**
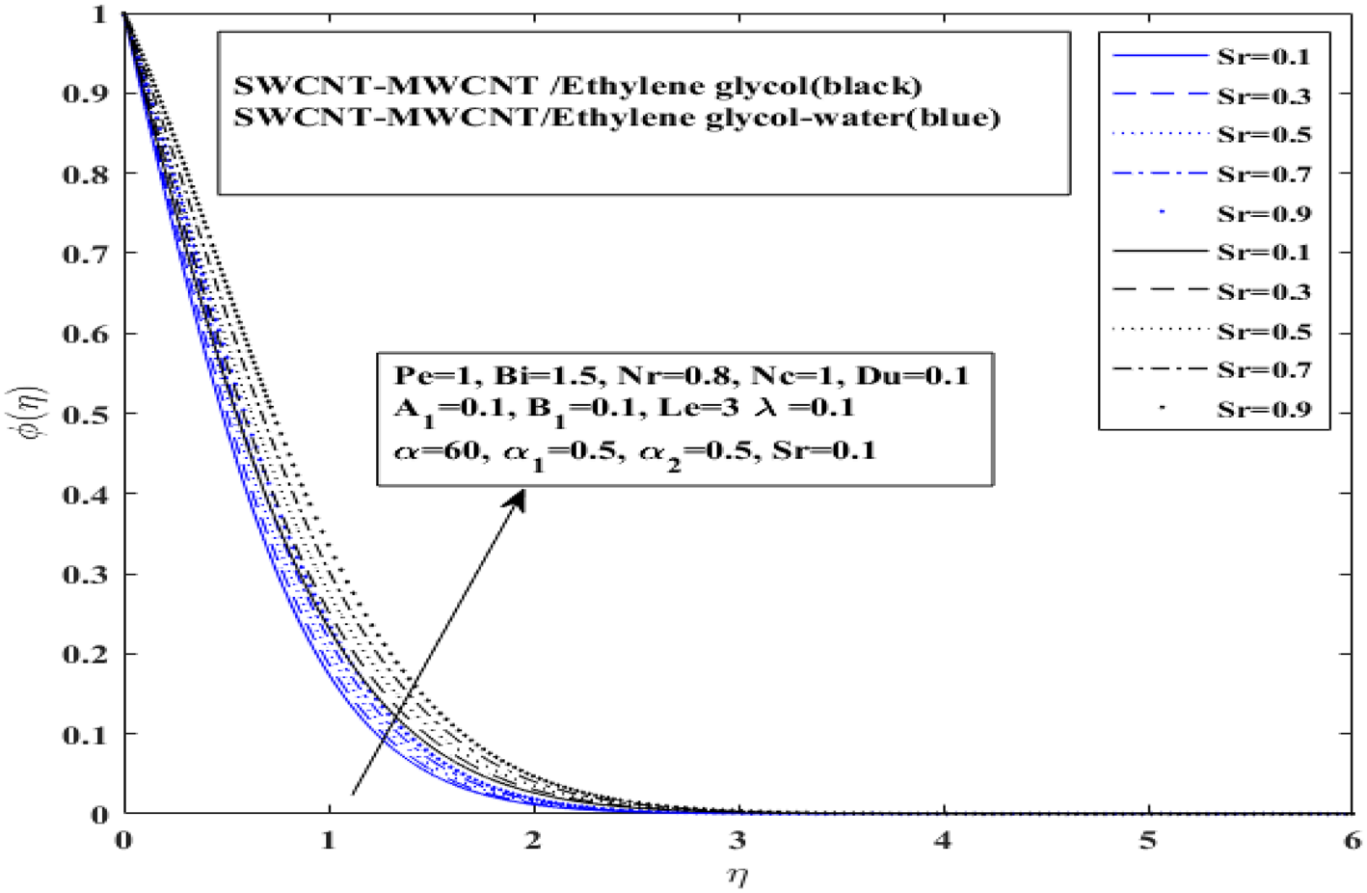
Impact of Soret number $$ Sr$$ influence on $$\varphi$$.

**Figure 13:**
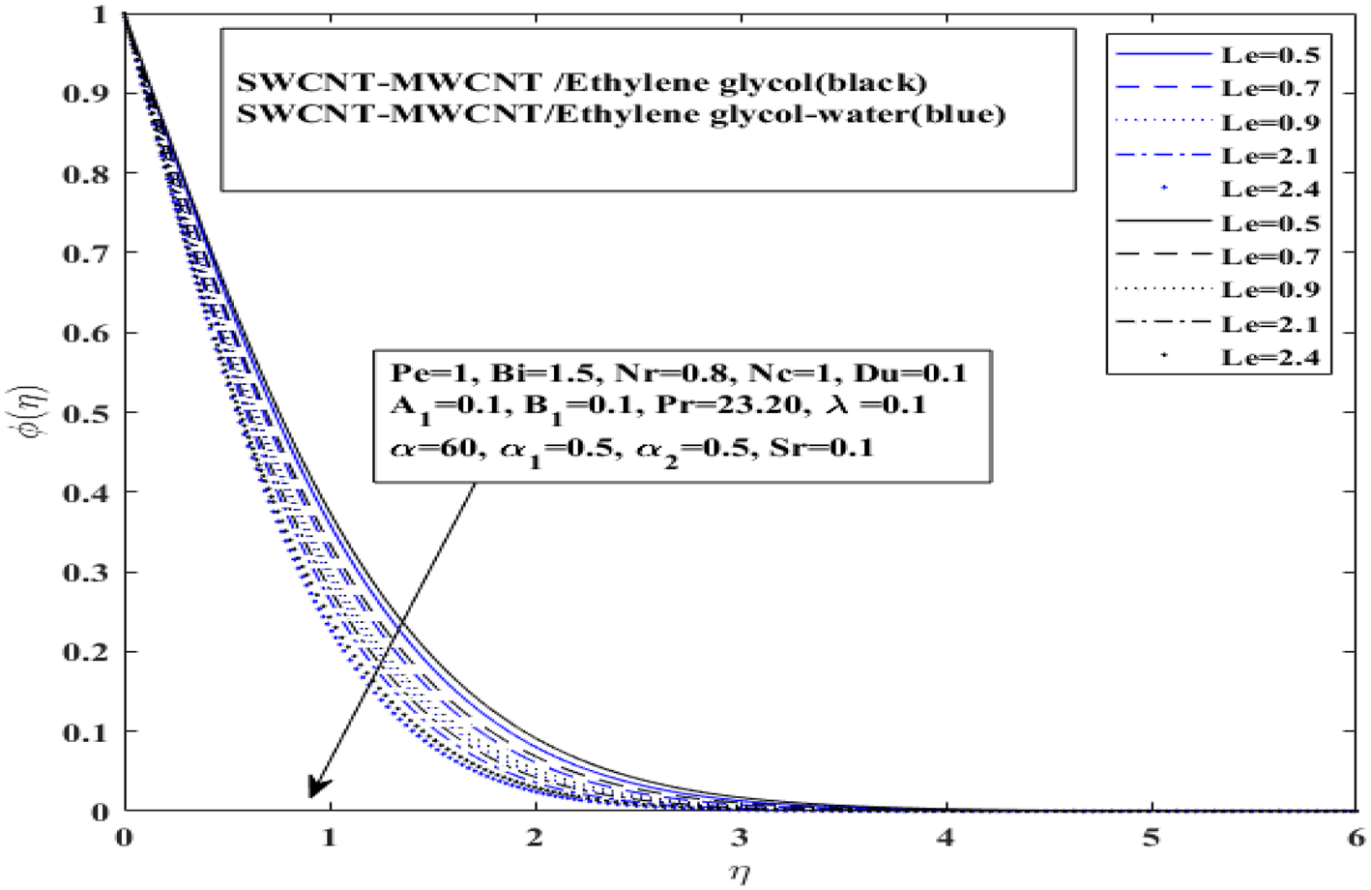
Impact of Lewis number $Le$ influence on $\varphi$.

The affect of $Lb$ (Bioconvection Lewis number) and $\left( {Pe} \right)$(Peclet number) over motile microorganism profile ($X(\eta$)) for $\left( {{\text{SWCNT}} - {\text{MWCNT}}/{\text{EG and SWCNT}} - {\text{MWCNT}}/{\text{EG}} - {\text{H}}_{2} {\text{O}}} \right)$ hybrid nanofluids in Figs. 10 and 11, respectively. Additionally, Soret effect (Sr) and Lewis number (*Le*) influence on concentration profile $\varphi \left( \eta \right)$ for both hybrid nanofluids $\left( {{\text{SWCNT}} - {\text{MWCNT}}/{\text{EG and SWCNT}} - {\text{MWCNT}}/{\text{EG}} - {\text{H}}_{2} {\text{O}}} \right)$ have been presented in Figs. 12 and 13, respectively. Bioconvection Lewis number impact has been observed on microorganisms profile in Fig. 10 and Peclet number impact has been observed on microorganisms profile in Fig. 11, for both type of hybrid nanofluids $\left( {{\text{SWCNT}} - {\text{MWCNT}}/{\text{EG and SWCNT}} - {\text{MWCNT}}/{\text{EG}} - {\text{H}}_{2} {\text{O}}} \right).$

The microorganism profiles are discovered to be enhanced by raising the Pe and decreases for Bioconvection Lewis number for both $\left( {{\text{SWCNT}} - {\text{MWCNT}}/{\text{EG and SWCNT}} - {\text{MWCNT}}/{\text{EG}} - {\text{H}}_{2} {\text{O}}} \right)$ hybrid nanofluids. It indicated that the $\left( {{\text{SWCNT}} - {\text{MWCNT}}/{\text{EG}}} \right)$ hybrid nanofluid microorganisms curve drops more quickly than the $\left( {{\text{SWCNT}} - {\text{MWCNT}}/{\text{EG}} - {\text{H}}_{2} {\text{O}}} \right)$ hybrid nanofluid microorganisms curve for Bioconvection Lewis number. Whereas, $\left( {{\text{SWCNT}} - {\text{MWCNT}}/{\text{EG}}} \right)$ hybrid nanofluid microorganisms curve grows more quickly than the $\left( {{\text{SWCNT}} - {\text{MWCNT}}/{\text{EG}} - {\text{H}}_{2} {\text{O}}} \right)$ hybrid nanofluid microorganisms curve for Peclet number. It is clear that shear expansion and flattening cause a drop in the microorganism profile. This is mostly due to a decline in the microorganism's diffusivity, which also affects its speed. Peclet number enhances the ratio of organisms, causing a boost in flow of fluid with expanding values.

The concentration profile of both $\left( {{\text{SWCNT}} - {\text{MWCNT}}/{\text{EG and SWCNT}} - {\text{MWCNT}}/{\text{EG}} - {\text{H}}_{2} {\text{O}}} \right)$ hybrid nanofluids is discussed in Figs. 12 and 13 under the effect of Soret number and Lewis number, respectively. The effect of a Soret number parameter on concentration profile is shown in Fig. 12 for $({\text{SWCNT}} - {\text{MWCNT}}/{\text{EG and SWCNT}} - {\text{MWCNT}}/{\text{EG}} - {\text{H}}_{2} {\text{O}}$). With a rise in the Soret number parameter, the concentration proficiency has grown. It is important to note that the concentration layer has grown as the Soret number parameter has increased. It is also interesting to note here that hybrid nanofluid ${\text{SWCNT}} - {\text{MWCNT}}/{\text{EG}} - {\text{H}}_{2} {\text{O}})$ with higher thermal conductivity does not guarantee for a higher migration of microorganisms when surface is heated and strong Soret effect is applied. Basically, Soret effect is the reverse process of Dufour effect.

The influence of Lewis number shows in Fig. 13 for $\left( {{\text{SWCNT}} - {\text{MWCNT}}/{\text{EG and SWCNT}} - {\text{MWCNT}}/{\text{EG}} - {\text{H}}_{2} {\text{O}}} \right)$. The concentration level has significantly fall when the Lewis number is enhanced. Furthermore, it indicated that the $\left( {{\text{SWCNT}} - {\text{MWCNT}}/{\text{EG}}} \right){ }$ nanofluid concentration curve drops more slowly than the ${\text{SWCNT}} - {\text{MWCNT}}/{\text{EG}} - {\text{H}}_{2} {\text{O}}){ }$ hybrid nanofluid concentration curve for Lewis number and grows more quickly for Soret number parameter. Moreover, a slight difference has been observed in concentration profile for both types of fluids when Lewis number is enhanced.

### Results of skin drag and heat transfer coefficients

Tables 4 and 5 illustrates the impact of inclined magnetization $0.1 \le {\text{M}} \le 0.5$, mixed convection $0.1 \le {\uplambda } \le 0.5$, thermal radiation $0.1 \le {\text{Rd}} \le 0.3$, Dufour effect $0.2 \le {\text{Du}} \le 0.4$, Soret effect $0.1 \le {\text{Sr}} \le 0.3$ and Lewis number $0.5 \le {\text{Le}} \le 2.4$ has been discussed comparatively for $\left( {{\text{SWCNT}} - {\text{MWCNT}}/{\text{EG}} - {\text{H}}_{2} {\text{O}}} \right)$ and $\left( {{\text{SWCNT}} - {\text{MWCNT}}/{\text{EG}}} \right){ }$ hybrid nanofluids for different values of parameters, respectively.

**Table 4:** Nusselt and skin friction coefficient for ${ }\left( {{\text{SWCNT}} - {\text{MWCNT}}/{\text{EG}} - {\text{H}}_{2} {\text{O}}} \right)$ hybrid nanofluid.

$$M$$	$$Rd$$	$$Du$$	$$ \lambda$$	$$Bi$$	$$Sr$$	$$ C_{fx} R_{{e_{x} }}^{0.5}$$	$$ Nu_{x} R_{{e_{x} }}^{ - 0.5}$$
0.1	0.1	0.2	0.1	0.5	0.1	− 2.3145	1.5577
0.3						− 2.2687	1.5584
0.5						− 2.2239	1.5591
0.1	0.1					− 2.3145	1.5577
	0.2					− 2.3111	1.4732
	0.3					− 2.3081	1.4006
	0.1	0.2				− 2.1926	1.3060
		0.3				− 2.2713	1.0561
		0.4				− 2.2506	0.8081
		0.2	0.1			− 2.3145	1.5577
			0.3			− 0.6548	1.5248
			0.5			− 0.1789	1.4794
			0.1	0.5		− 2.6393	0.01827
				1.0		− 2.4820	0.23880
				1.5		− 2.3984	0.34013
				0.5	0.1	− 2.3145	1.5577
					0.2	− 2.2915	1.6196
					0.3	− 2.2676	1.6836

**Table 5:** Nusselt and skin friction coefficient for $\left( {{\text{SWCNT}} - {\text{MWCNT}}/{\text{EG}}} \right)$ hybrid nanofluid.

$$M$$	$$Rd$$	$$Du$$	$$ \lambda$$	$$Bi$$	$$Sr$$	$$ C_{fx} R_{{e_{x} }}^{0.5}$$	$$ Nu_{x} R_{{e_{x} }}^{ - 0.5} Nu_{x} R_{{e_{x} }}^{ - 0.5}$$
1.0	0.1	0.2	0.1	0.5	0.1	− 2.8515	1.5817
1.3						− 2.7593	1.6349
1.5						− 2.7112	1.7460
1.0	0.1					− 2.8515	1.5817
	0.2					− 2.7416	1.9425
	0.3					− 2.6952	2.6614
	0.1	0.2				− 2.8515	1.5817
		0.3				− 2.7917	1.5303
		0.4				− 2.7581	0.6827
		0.2	0.1			− 2.8515	1.5817
			0.3			− 0.6423	0.4403
			0.5			− 0.7723	0.0184
			0.1	0.5		− 2.8515	0.0928
				1.0		− 2.6634	0.8809
				1.5		− 2.4112	1.5889
				0.5	0.1	− 2.8515	1.5817
					0.2	− 2.7658	1.0344
					0.3	− 0.7005	0.5261

The outcomes for the mixture base hybrid nanofluid ${ }\left( {{\text{SWCNT}} - {\text{MWCNT}}/{\text{EG}} - {\text{H}}_{2} {\text{O}}} \right)$ have been presented in Table 4 under varying impact of study parameters. It has been observed that with higher mixed convection levels higher skin friction coefficient has been obtained for mixture based hybrid nanofluid whereas reduced or minimum skin friction on the heated surface has been observed under the effect of varying thermal Biot number. Furthermore, magnetization, thermal radiation, Dufour and Soret number produce higher skin surface for mixture base hybrid nanofluid. Additionally, the maximum Nusselt number has been observed under the thermal radiation impact whereas minimum rates of heat transfer has been observed by increasing the Dufour effect levels.

The numerical outcomes for single base hybrid nanofluid $\left( {{\text{SWCNT}} - {\text{MWCNT}}/{\text{EG}}} \right)$ has been presented under the influence of study parameters in Table 5. We noticed that skin friction further increase by increasing the different study parameters whereas the Nusselt number enhance under the varying thermal radiation, thermal Biot and Soret effect. Higher rates of heat transfer has been observed with increasing thermal radiation effect.

## Conclusions

In this article comparative dynamics of hybrid nanofluids $\left( {{\text{SWCNT}} - {\text{MWCNT}}/{\text{EG and SWCNT}} - {\text{MWCNT}}/{\text{EG}} - {\text{H}}_{2} {\text{O}}} \right)$ have been investigated incorporating the carbon single wall and multi wall as nanoparticles for heat transfer features of bioconvective flow over heated stretched surface. Non- Newtonian Prandtl fluid model has been utilized considering microorganisms in the presence of convective boundary condition and thermal radiation effect. Additionally, Soret and Dufour effects have also been taken into account and impact of these effects have been analyzed. The microorganism regime over heated surface is employed to achieve bioconvective flow regime for Prandtl hybrid nanofluids. Mathematical model had been developed to incorporate all the mentioned physics and similarity transform had been used to achieve dimensionless flow governing equations. Results had been obtained using bvp-4c methodology in MATLAB. Following are the major outcomes of the present analysis:

It has been noted that with increment in Dufour effect temperature profile for hybrid nanofluids $\left( {{\text{SWCNT}} - {\text{MWCNT}}/{\text{EG and SWCNT}} - {\text{MWCNT}}/{\text{EG}} - {\text{H}}_{2} {\text{O}}} \right)$ enhance over heated surface when strong magnetization force is applied. Additionally, Soret effect improves concentration profile of both hybrid nanofluids $\left( {{\text{SWCNT}} - {\text{MWCNT}}/{\text{EG and SWCNT}} - {\text{MWCNT}}/{\text{EG}} - {\text{H}}_{2} {\text{O}}} \right)$ when magnetization angle is kept at 60 degrees.

Heat transfer coefficient decrease when level of Dufour effect are enhanced for both hybrid nanofluids $\left( {{\text{SWCNT}} - {\text{MWCNT}}/{\text{EG and SWCNT}} - {\text{MWCNT}}/{\text{EG}} - {\text{H}}_{2} {\text{O}}} \right)$. Whereas, enhancing Soret effect levels higher heat transfer coefficient has been observed for mixture base hybrid nanofluid. Furthermore, a reverse behavior has been observed for single base hybrid nanofluid.

Enhancing thermal radiation levels improves temperature profile significantly for hybrid nanofluid $\left( {{\text{SWCNT}} - {\text{MWCNT}}/{\text{EG}} - {\text{H}}_{2} {\text{O}}} \right)$ with higher thermal conductivity as compared to hybrid nanofluid $\left( {{\text{SWCNT}} - {\text{MWCNT}}/{\text{EG}}} \right)$ with less thermal conductance. Moreover, temperature dependent and space dependent heat source enhance temperature profile comparatively at same level for both types of hybrid nanofluid $\left( {{\text{SWCNT}} - {\text{MWCNT}}/{\text{EG and SWCNT}} - {\text{MWCNT}}/{\text{EG}} - {\text{H}}_{2} {\text{O}}} \right)$.

Inclined magnetization expand thickness of boundary layer for both hybrid nanofluids $\left( {{\text{SWCNT}} - {\text{MWCNT}}/{\text{EG and SWCNT}} - {\text{MWCNT}}/{\text{EG}} - {\text{H}}_{2} {\text{O}}} \right)$. Higher magnetization produce higher fluid acceleration irrespective of fluid type. Mixed convection decrease boundary layer thickness, whereas a severe contraction has been observed when inclination angle is improved gradually.

Reduced skin friction coefficient has been obtained for both types of fluid $\left( {{\text{SWCNT}} - {\text{MWCNT}}/{\text{EG and SWCNT}} - {\text{MWCNT}}/{\text{EG}} - {\text{H}}_{2} {\text{O}}} \right)$ by raising the values of mixed convection, inclined magnetization, Dufour, Soret and thermal Biot number. On the other hand raising levels of thermal radiation, mixed convection and thermal Biot number enhance Nusselt number significantly.

## Data Availability

The datasets used and/or analysed during the current study available from the corresponding author on reasonable request.

## List of symbols

*C*: Dimensionless concentration
*N*: Motile microorganism’s density
*T*: Dimensionless temperature
*C*_*w*_: Fluid concentration at surface
$N_{w} , N_{\infty }$: Ambient and surface microorganism’s density
*T*_*∞*_: Ambient temperature
*C*_*∞*_: Ambient concentration
*A*_*1*_: Space-dependent Heat Source/Sink
$\lambda$: Mixed conviction Parameter
$N_{r} and N_{c}$: Buoyancy ratio and bioconvection Rayleigh Number
*T*_*w*_: Fluid Temperature at surface
$u_{w}$: Stretching velocity (ms^−1^)
$\mu_{f}$: Kinematic viscosity (Nsm^−2^)
*B*_*1*_: Temperature dependent Heat Source/Sink
*D*_*2*_: Mass diffusion coefficient
$\alpha_{1}$: Prandtl fluid parameter
($$u$$,$$ v$$): Velocity components (ms^−1^)
*B*_*0*_: Magnetization force
$\omega , \omega_{c}$: Bioconvection constant and maximum cell swimming velocity (ms^−1^)
D_1_: Temperature diffusion coefficient
$${\upalpha }$$: Inclined angle
$C_{{{\varvec{fx}}}}$: Skin friction along X-direction
$\alpha_{hnf}$: Thermal diffusivity of HNF (m^−2^ s^−1^)
*Re*_*x*_: Reynolds Number
*Q'''*: Non uniform heat source/sink
*D*_*m*_: Microorganism coefficient
$${\upalpha }_{2}$$: Elasticity parameter
Sr: Soret number
$q_{c}$: Radiative heat flux
$$Du$$: Dufour number
$\rho_{m}$: Motile microorganism density (Kgm^−3^)
*D*_*B*_: Brownian diffusion coefficient
*Pr*: Prandtl number
*Rd*: Radiation parameter
$$\eta$$: Dimensionless variable
*Lb*: Bioconvection Lewis number
SWCNT/MWCNT: Single and multi wall carbon nanotubes
*Nu*_*x*_: Nusselt number
$Bi,Le$: Thermal Biot and Lewis number
$\rho_{f}$(Kgm^−3^): Density of Nanofluids
$M, Pe$: Magnetic and Peclet number
$\mu_{hnf}$: Kinematic viscosity of HNF (Nsm^−2^)
